# Evaluation of multiple sclerosis severity using a new OCT tool

**DOI:** 10.1371/journal.pone.0288581

**Published:** 2023-07-13

**Authors:** Elisa Viladés, Beatriz Cordón, Javier Pérez-Velilla, Elvira Orduna, Maria Satue, Vicente Polo, Berta Sebastian, Jose Manuel Larrosa, Luis Pablo, Elena García-Martin

**Affiliations:** 1 Miguel Servet Ophthalmology Research and Innovation Group (GIMSO), Aragon Institute for Health Research (IIS Aragón), University of Zaragoza, Zaragoza, Spain; 2 Ophthalmology Department, Miguel Servet University Hospital, Zaragoza, Spain; 3 Neurology Department, Miguel Servet University Hospital, Zaragoza, Spain; Icahn School of Medicine at Mount Sinai, UNITED STATES

## Abstract

**Purpose:**

To assess the ability of a new posterior pole protocol to detect areas with significant differences in retinal nerve fiber layer (RNFL) and ganglion cell layer (GCL) thickness in patients with multiple sclerosis versus healthy control subjects; in addition, to assess the correlation between RNFL and GCL thickness, disease duration, and the Expanded Disability Status Scale (EDSS).

**Methods:**

We analyzed 66 eyes of healthy control subjects and 100 eyes of remitting-relapsing multiple sclerosis (RR-MS) patients. Double analysis based on first clinical symptom onset (CSO) and conversion to clinically definite MS (CDMS) was performed. The RR-MS group was divided into subgroups by CSO and CDMS year: CSO-1 (≤ 5 years) and CSO-2 (≥ 6 years), and CDMS-1 (≤ 5 years) and CDMS-2 (≥ 6 years).

**Results:**

Significant differences in RNFL and GCL thickness were found between the RR-MS group and the healthy controls and between the CSO and CDMS subgroups and in both layers. Moderate to strong correlations were found between RNFL and GCL thickness and CSO and CDMS. Furthermore, we observed a strong correlation with EDSS 1 year after the OCT examination.

**Conclusions:**

The posterior pole protocol is a useful tool for assessing MS and can reveal differences even in early stages of the disease. RNFL thickness shows a strong correlation with disability status, while GCL thickness correlates better with disease duration.

## Introduction

Although multiple sclerosis (MS) has traditionally been considered a rare disease, it is now the most common chronic autoimmune, demyelinating, and neurodegenerative disease of the central nervous system (CNS) and, after traumatic injury, is the second most common cause of permanent disability among young adults [1].

In 2019 it was estimated that there were 2.2 million people with MS worldwide [2]. Furthermore, several countries have observed significant increases in MS incidence, changes in the male:female ratio, or new pediatric cases [3–5].

In the last decade, studies have widely demonstrated that the anterior visual pathway provides information about the dynamics of axonal degeneration in MS caused by episodes of acute optic neuritis, subclinical optic neuropathy, and/or retrograde degeneration [6–10].

The retina is an accessible and visible "window to the brain" that provides *in vivo* information about the CNS via non-invasive imaging techniques such as optical coherence tomography (OCT). Previous studies have suggested that axons and neurons in the retina can be quantified by spectral-domain OCT [9–14]. The peripapillary retinal nerve fiber layer (pRNFL), composed of unmyelinated axons, has been suggested as a biomarker representing axonal degeneration in the brain of MS patients. Given OCT’s very high degree of reproducibility, it offers a potential means of tracking neuroaxonal loss in MS at individual patient level [6–8].

Monitoring of early degeneration and disease progression, as well as treatment success, may all be enhanced by analyzing axonal microstructure integrity [6]. However, monitoring pRNFL thickness may not be as accurate at detecting early alterations in axonal microstructure. Previous cross-sectional studies using SD-OCT have shown macular ganglion cell + inner plexiform layer (mGCIPL) thickness to offer greater reliability and repeatability, as well as better connection with visual function and clinical disability in MS, than pRNFL thickness [7–15]. Although retinal injury worsens as the disease progresses [16, 17], retinal axonal loss has been noted at initial onset of the disease. Development of the algorithms used by SD-OCT software enables more precise segmentation of the various retinal layers and pinpoints potential abnormalities.

The mGCIPL thinning rate is accelerated in MS patients exhibiting inflammatory activity [18] and correlates strongly with brain atrophy, particularly gray matter atrophy, over time. A large-scale pathology study concluded that retinal injury includes not only axonal loss (thinning of the pRNFL), but also a reduction in ganglion cell density, demonstrating that inner retinal thinning in MS reflects neuronal and axonal loss [7–16]. Detecting the relationship between the different types of retinal injury in MS might help us understand which factors drive both inflammatory and tissue atrophy.

The objective of this study is to analyze the retinal nerve fiber layer (RNFL) and ganglion cell layer (GCL) in MS patients using a new software protocol designed for SD-OCT: the posterior pole protocol. Previous studies conducted using this new protocol have mainly addressed glaucoma [18] and healthy Caucasian populations [19, 20]. The findings showed that in early-stage glaucoma, clusters of sectors in the GCL provided better sensitivity and specificity values than clusters in the pRNFL [21]. However, the posterior pole protocol’s ability to detect changes in pRNFL and GCL thickness in MS patients has not been studied yet. The McDonald Criteria must be used to correctly diagnose MS because time plays a significant role in the disease and is linked to subsequent deterioration and impairment. The criteria indicate that in order to diagnose MS, there must be evidence of damage separated in time and space, with lesions in two independent areas of the CNS. These tests attempt to make an early diagnosis of MS while maintaining high sensitivity and specificity.

Our study aims to evaluate the correlation between disease duration and the damage that occurs in the retina and so predict the future disability of the patient.

## Methods

All procedures in this study adhered to the tenets of the Declaration of Helsinki; the experimental protocol was approved by the Ethics Committee of Miguel Servet University Hospital (CEICA), and all participants provided written informed consent to participate in the study.

We included patients with definite relapsing-remitting MS (RR-MS), diagnosed according to the 2010 revision of the McDonald Criteria and confirmed by a specialized neurologist [22]. Patients were assigned to the clinically definite multiple sclerosis (CDMS) group based on the date they met the criteria, or to the clinical symptom onset (CSO) group based on the date that symptoms were identified but did not yet meet the MS diagnosis criteria. The Expanded Disability Status Scale (EDSS) scores at OCT examination (EDSS-0) and 1 year later (EDSS-1) were collected from neurological examinations.

A total of 100 eyes from 100 MS patients and 66 eyes from 66 healthy individuals were evaluated by a neuro-ophthalmologist. One eye per subject was randomly selected. In the RR-MS group, the eye without previous episodes of optic neuritis was randomized and eyes with previous episodes of optic neuritis were excluded to avoid bias when assessing the extent to which neurodegeneration is detectable in retinal layers. Eyes longer than 25.2 mm or with refractive errors ≥ 5 diopters (D) of spherical equivalent or ≥ 3 D of astigmatism were excluded from the study. In addition, ophthalmological examination was used to detect ocular alterations such as macular or optic disc damage, cataract, or media opacity that could affect functional vision or captured images.

We classified the 100 RR-MS patients from the main group into two subgroups based on time of diagnosis of CDMS for the first analysis, and created another two subgroups based on first CSO for the second analysis. The latter were divided into CSO-1 (≤ 5 years) comprising 38 eyes from 38 individuals and CSO-2 (≥ 6 years) comprising 62 eyes from 62 subjects; and CDMS-1 (≤ 5 years) comprising 50 eyes from 50 individuals and CDMS-2 (≥ 6 years) comprising 49 eyes from 49 subjects.

**Fig 1:**
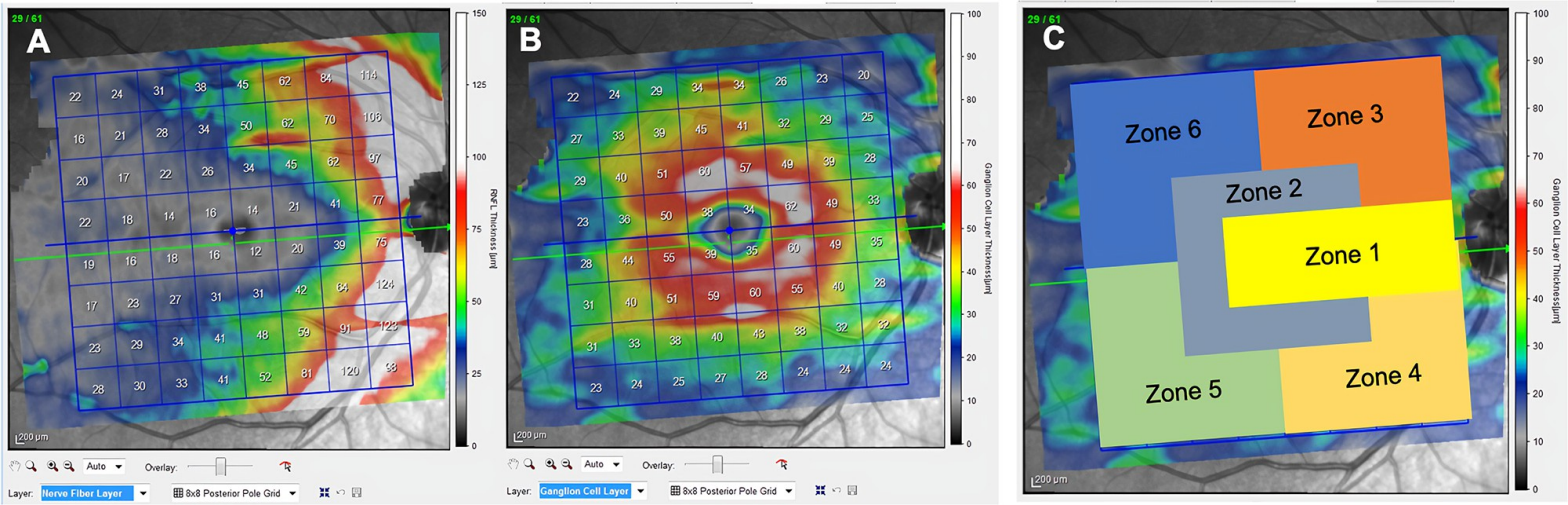
Image of posterior pole protocol. A: Color thickness map of the 64 OCT scans and the 8 × 8 analysis grid positioned on the macula for the RNFL; B: Color thickness map of the 64 OCT scans and the 8 × 8 analysis grid positioned on macula for the GCL; numerical values on the grid indicate microns of thickness for each cell. The color map associates warm colors with higher thickness values and cool colors with lower thickness values in a gradient, indicating the value in the legend every 25 microns (RNFL. Image A) and every 10 microns (GCL. Image B); C: six proposed zones on the macular thickness map, labeled with different colors.

Structural measurements of the retina were obtained using the Spectralis OCT device (Heidelberg Engineering, Germany). The posterior pole protocol was used for all subjects (Fig 1). This protocol allows for detailed segmentation of the retinal layers. It incorporates the Anatomic Positioning System (APS), which describes a horizontal line between the fovea and the Bruch membrane opening. Based on that reference line, 61 parallel explorations are performed inside a 25º × 30º area. APS plus the True Track eye-tracking system ensure accurate detection of macula position in each individual based on head tilt and eye cyclotorsion. All measurements were taken by a single operator blind to group classification. Low-quality images (quality score below 25/40) and images with movement artifacts were excluded from the analysis. This 25º × 30º area is divided into an 8 × 8 grid, which provides global retinal thickness and segments the thickness of each layer into 64 independent cells. The Spectralis OCT device has an axial (in tissue) resolution of 3.9 μm and the thickness value is obtained from the average of each cell (1 × 1 mm). This protocol also provides a color map scale for visual changes of 10–15 μm, thus permitting improved detection of small tissue thickness losses by visual inspection of the retinal thickness map. In this study, we evaluated the RNFL and the GCL.

No manual correction was applied to the OCT output. An internal fixation target was used because it is reported to have the highest reproducibility [23]. The quality of the scans was assessed prior to the analysis and poor-quality scans were rejected. The Spectralis OCT device uses a blue quality bar in the image to indicate signal strength. The quality score ranges from 0 (poor quality) to 40 (excellent quality). Based on previously published studies on the effect of image quality on tissue thickness measurements using similar devices, only images with quality scores above 25 were included in the analysis [24]. Due to the number of cells with data, and to better visualize and understand the information, descriptive data from 64 measured cells were grouped into 6 zones. RNFL and GCL measurements were recorded in a database and analyzed with the IBM-SPSS Statistics package (SPSS Inc., Chicago, IL, USA, version 20.0).

The Kolmogorov–Smirnov test identified the study variables as normally distributed. As the RNFL data were parametric, Student’s *t*-test was used to compare the controls and the MS patients, and the one-way ANOVA test was used to perform multiple comparisons between the subgroups. As the GCL data were not parametric, the Mann–Whitney *U* test was used to compare the control and MS cohorts, and the one-way ANOVA Kruskal–Wallis *H* test was used to perform multiple comparisons between the subgroups. For correlation, the Pearson correlation coefficient was used for the RNFL and the Spearman correlation coefficient was used for the GCL. We then analyzed the correlations between visual acuity, MS severity measured by EDSS, and retinal thickness (using the average of the RNFL and the GCL).

**Fig 2:**
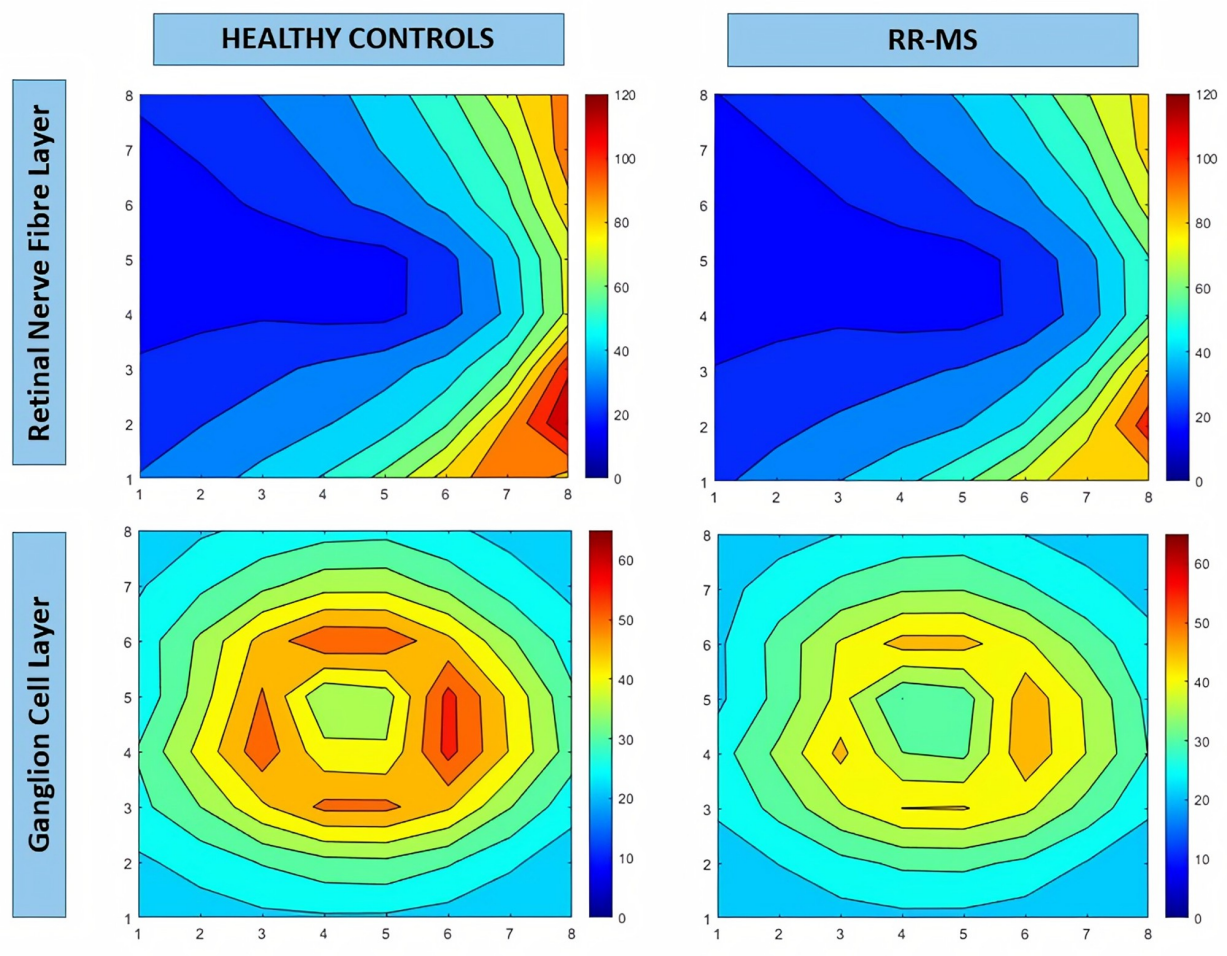
Contour map, generated from numerical data on each cell measured using the posterior pole protocol, showing mean retinal fiber layer thickness analysis in Mathworks. The legend indicates 10-micron thickness steps, with warm colors representing higher thickness values and cool colors representing lower thickness values. A: retinal fiber layer from control group; B: retinal fiber layer from remitting-relapsing multiple sclerosis; C: ganglion cell layer from control group; D: ganglion cell layer from remitting-relapsing multiple sclerosis. Abbreviations: RR-MS, remitting-relapsing multiple sclerosis.

**Fig 3:**
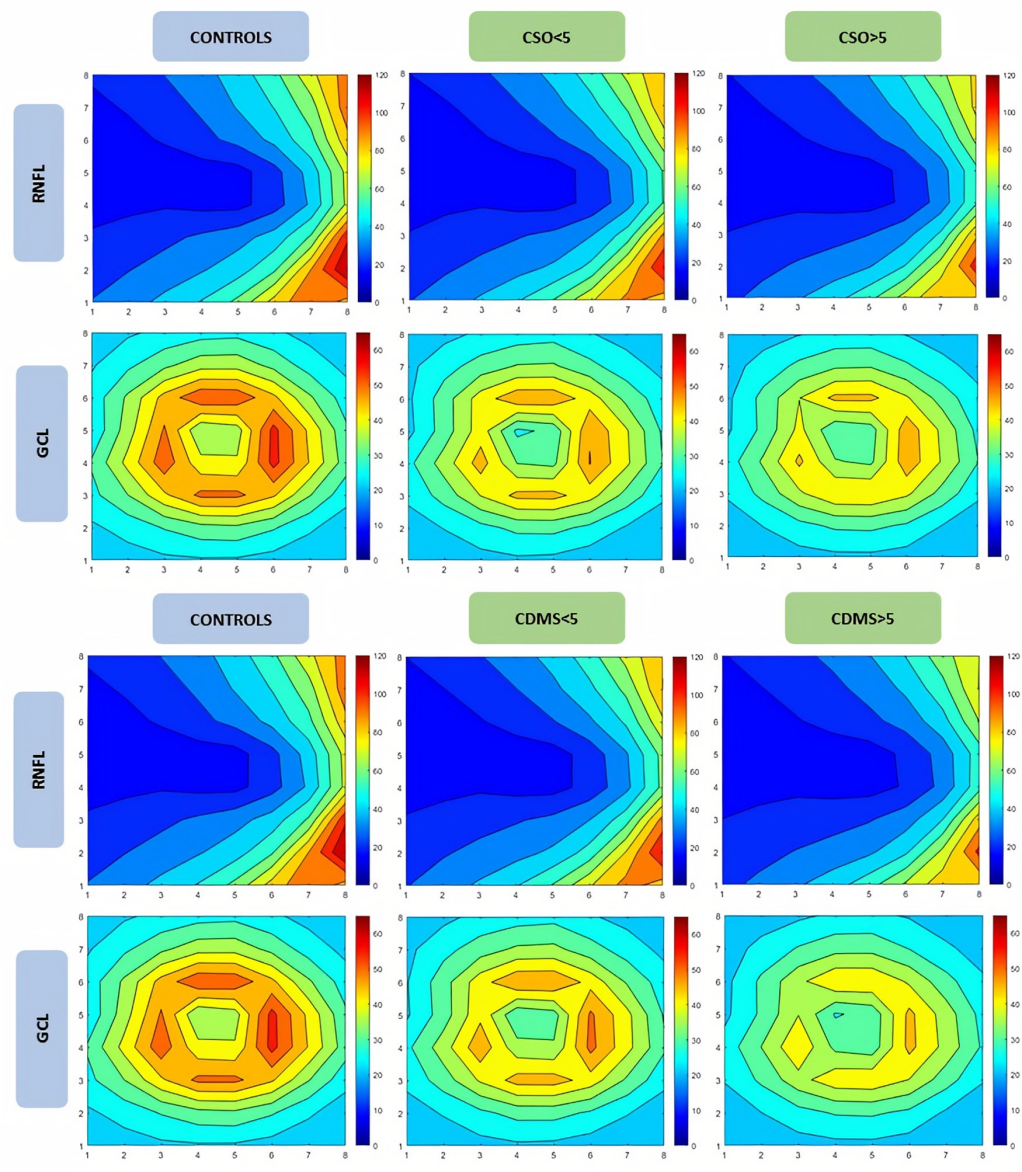
Contour map, generated from numerical data on each cell measured using the posterior pole protocol, showing mean retinal fiber layer and ganglion cell layer thickness analysis in Mathworks for subgroups of years. The legend associates warm colors with higher thickness values in microns and cool colors with lower thickness values. The upper images show clinical symptom onset analysis and the lower images show clinically definite multiple sclerosis. Abbreviations: CSO, clinical symptom onset; CDMS, clinically definite multiple sclerosis; RNFL, retinal nerve fiber layer; GCL, ganglion cell layer.

**Fig 4:**
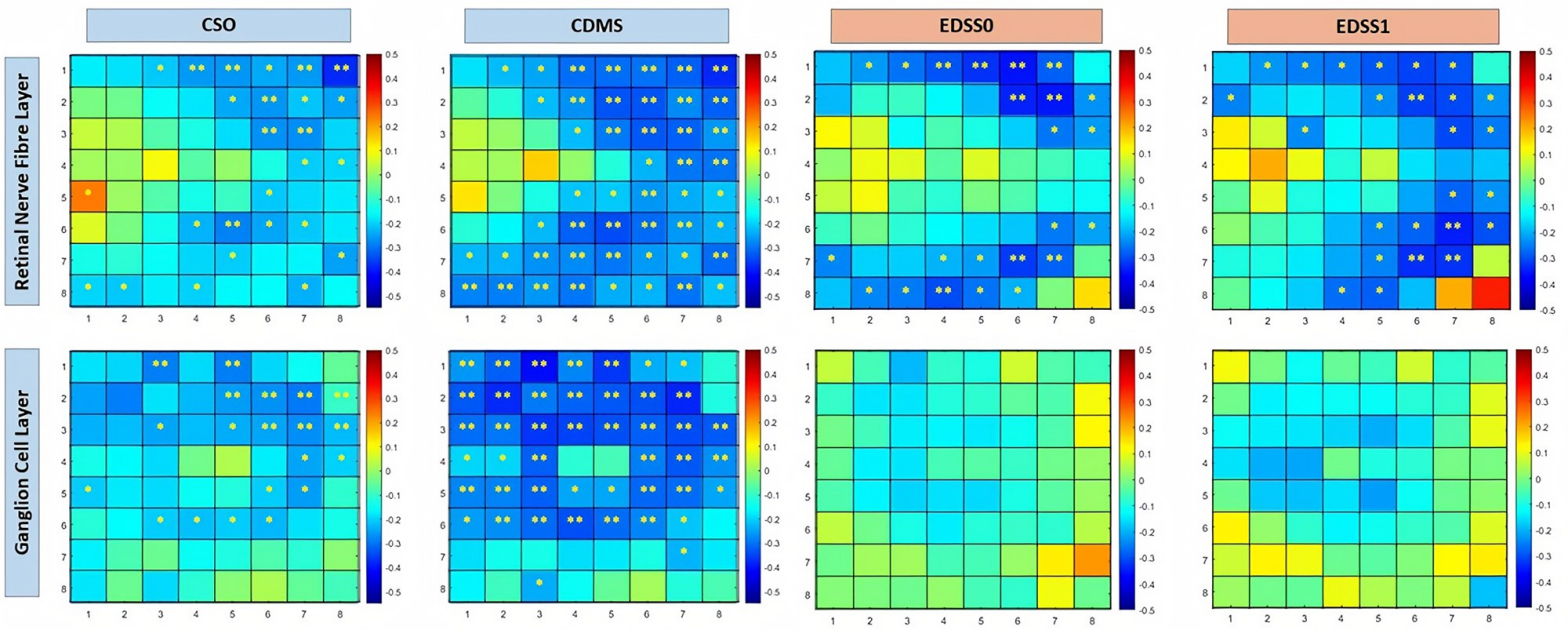
Image of numerical data of mean Pearson correlation coefficient analysis for the RNFL and Spearman correlation coefficient for the GCL in Mathworks. Each map represents the grid of 64 pole cells, with color depending on the correlation value indicated in the legend in steps from 0.1 associated with a color. Positive correlation values are associated with warm colors and negative correlation values are associated with cool colors. Correlation between the retinal nerve fiber layer and time since symptom onset, time since clinically definite multiple sclerosis diagnosis, expanded disability status scale at optical coherence tomography examination, and 1 year after; correlation between the ganglion cell fiber layer and time since symptom onset, time since clinically definite multiple sclerosis, expanded disability status scale at optical coherence tomography examination, and 1 year after. The asterisks show significant levels that overcome Bonferroni correction for multiple comparisons. * p < 0.05, ** p ≤ 0.005. Abbreviations: CSO, clinical symptom onset; CDMS, clinically definite multiple sclerosis; EDSS-0, expanded disability status scale at optical coherence tomography examination; EDSS-1, expanded disability status scale 1 year after optical coherence tomography examination.

The numerical data obtained with SPSS were analyzed in MATLAB (R2020a, Mathworks, Massachusetts, USA) [25], which allows matrix representation using the M language. This program has previously been used in biological tissue research and in ophthalmic research [26–29]. Since posterior pole protocol analysis is based on an 8 × 8 grid, which works as a matrix, numerical data can be reassembled and processed in MATLAB. The mean RNFL and GCL thicknesses from 64 cells were represented on a contour map (Fig 2); taking the p-values obtained from the one-way ANOVA test and the Pearson and Spearman correlation coefficients, we obtained image plots (Figs 3 and 4, respectively).

## Results

In the control group, mean age was 44.52 ± 18.91 years, intraocular pressure (IOP) was 13.67 ± 2.38 mm Hg, and gender distribution was 17 males (25.8%) and 49 females (74.2%). In the MS group, mean age was 41.94 ± 13.86 years, IOP was 13.68 ± 2.10 mm Hg, gender distribution was 20 males (20.0%) and 80 females (80.0%), mean EDSS score was 2.39 ± 1.73 (range: 0–7.5), and 1 year after OCT acquisition mean EDSS score was 2.43 ± 2.17 (range: 0–7.5).

Mean visual acuity was 0.91 ± 0.23 in the control group and 0.85 ± 0.33 in the MS group. We did not find any correlation between visual acuity and MS severity measured by EDSS (r = 0.611; p = 0.452) or between mean RNFL (r = 0.589; p = 0.101) and GCL thickness (r = 0.515; p = 0.277).

### Retinal nerve fiber layer

Patients with RR-MS presented significant RNFL thinning versus controls (Table 1; Fig 2).

**Table 1:** Mean retinal nerve fiber and ganglion cell layer thickness ± standard deviation, in microns, of 6 zones for healthy controls and remitting-relapsing multiple sclerosis patients. Bold numbers indicate p < 0.05. Abbreviations: RNFL, retinal nerve fiber layer; SD, standard deviation; GCL, ganglion cell layer; RR-MM, remitting-relapsing multiple sclerosis.

		CONTROLS		RR-MS		Student’s *t*-test
		Mean *(μm)*	±SD	Mean *(μm)*	±SD	
**RNFL**	ZONE 1	35.01	3.75	29.79	5.36	**<0.001**
	ZONE 2	29.82	3.04	26.31	3.88	**<0.001**
	ZONE 3	67.39	8.99	59.79	12.17	**<0.001**
	ZONE 4	82.18	13.15	72.47	13.87	**<0.001**
	ZONE 5	30.77	4.73	28.83	5.15	**0.015**
	ZONE 6	23.15	3.59	22.19	4.57	0.156
**GCL**	ZONE 1	42.11	3.00	36.78	5.93	**<0.001**
	ZONE 2	49.95	3.74	43.70	6.97	**<0.001**
	ZONE 3	28.57	2.35	27.45	3.19	**0.015**
	ZONE 4	26.63	2.60	25.59	2.50	**0.011**
	ZONE 5	28.69	2.59	26.62	2.72	**<0.001**
	ZONE 6	29.38	2.61	27.11	3.25	**<0.001**

The one-way ANOVA test revealed significant differences in the CSO RR-MS subgroups versus healthy controls (Table 2) in 26 cells: zones 1, 2, 3, 4, and 5 in the RNFL and all zones in the GCL (Fig 3, upper).

**Table 2:** Mean retinal nerve fiber and ganglion cell layer thickness ± standard deviation, in microns, of 6 zones for remitting-relapsing multiple sclerosis patients classified by years since onset of symptoms and comparison between both groups. Bold numbers indicate p < 0.05. The asterisks mark significant levels that overcome Bonferroni correction for multiple comparisons. Abbreviations: RNFL, retinal nerve fiber layer; SD, standard deviation; GCL, ganglion cell layer; RR-MM, remitting-relapsing multiple sclerosis; CSO, clinical symptom onset.

		CONTROLS		CSO-1		CSO-2		One-way ANOVA
		n = 66		n = 38		n = 62		
		Mean *(μm)*	±SD	Mean *(μm)*	±SD	Mean *(μm)*	±SD	
**RNFL**	ZONE 1	35.01	3.75	30.71	4.94	29.22	5.57	*<0*.001*
	ZONE 2	29.82	3.04	27.28	3.40	25.72	4.05	*<0*.001*
	ZONE 3	67.39	8.99	62.57	9.08	58.08	13.51	*<0*.001*
	ZONE 4	82.18	13.15	74.99	10.81	70.92	15.26	*<0*.001*
	ZONE 5	30.77	4.72	29.74	4.02	28.27	5.69	***0*.*019***
	ZONE 6	23.15	3.59	22.08	2.28	22.26	5.55	0.360
**GCL**	ZONE 1	42.11	3.00	36.99	5.86	36.65	6.02	*<0*.001*
	ZONE 2	49.95	3.74	44.78	6.93	43.05	6.97	*<0*.001*
	ZONE 3	28.57	2.35	28.18	3.35	27.01	3.02	***0*.*011***
	ZONE 4	26.63	2.60	25.38	2.37	25.72	2.59	***0*.*030***
	ZONE 5	28.69	2.59	26.81	2.79	26.50	2.69	*<0*.001*
	ZONE 6	29.38	2.61	27.55	3.33	26.84	3.19	*<0*.001*

*Post hoc* Bonferroni analysis (Fig 3, upper) showed significant differences in subgroup CSO-1 versus healthy controls in zones 1 (p < 0.001), 2 (p = 0.002), and 4 (p = 0.030). In CSO-2, significant thinning was observed in zones 1 (p < 0.001), 2 (p < 0.001), 3 (p < 0.001), 4 (p < 0.001), and 5 (p = 0,015) versus healthy controls. No differences were found between the two CSO subgroups.

The one-way ANOVA test showed significant differences in the CDMS subgroups (Table 3, lower) in 27 cells. When the CDMS subgroups of MS patients were compared with healthy controls, *post hoc* analysis (Fig 3, lower) revealed significant differences versus healthy controls in CDMS-1: zones 1 (p < 0.001), 2 (p < 0.001), and 4 (p = 0.020). CDMS-2 showed significant differences in zones 1 (p < 0.001), 2 (p < 0.001), 3 (p < 0.001), 4 (p < 0.001), and 5 (p = 0.005). Significant differences were found in CDMS-1 and CDMS-2 between zones 1 (p = 0.011), 2 (p = 0.026), and 3 (p = 0.012).

**Table 3:** Mean retinal nerve fiber and ganglion cell layer thickness ± standard deviation, in microns, of 6 zones for remitting-relapsing multiple sclerosis patients classified by years since clinically definite multiple sclerosis diagnosis and comparison between both groups. Bold numbers indicate p < 0.05. The asterisks mark significant levels that overcome Bonferroni correction for multiple comparisons. Abbreviations: RNFL, retinal nerve fiber layer; SD, standard deviation; GCL, ganglion cell layer; RR-MM, remitting-relapsing multiple sclerosis; CDMS, clinically definite multiple sclerosis.

		CONTROLS		CDMS-1		CDMS-2		One-way ANOVA
		n = 66		n = 50		n = 49		
		Mean *(μm)*	±SD	Mean *(μm)*	±SD	Mean *(μm)*	±SD	
**RNFL**	ZONE 1	35.01	3.75	31.22	5.35	28.44	5.06	*<0*.001*
	ZONE 2	29.82	3.04	27.25	3.50	25.38	4.08	*<0*.001*
	ZONE 3	67.39	8.99	62.89	9.43	56.57	13.93	*<0*.001*
	ZONE 4	82.18	13.15	75.31	11.02	69.19	15.63	*<0*.001*
	ZONE 5	30.77	4.73	29.79	3.87	27.78	6.08	***0*.*007***
	ZONE 6	23.15	3.59	22.34	2.62	22.06	6.01	0.358
**GCL**	ZONE 1	42.11	3.00	38.15	5.89	35.29	5.69	*<0*.001*
	ZONE 2	49.95	3.74	45.59	6.72	41.63	6.69	*<0*.001*
	ZONE 3	28.57	2.35	28.43	3.25	26.37	2.76	*<0*.001*
	ZONE 4	26.63	2.60	25.92	2.59	25.18	2.34	***0*.*011***
	ZONE 5	28.69	2.59	27.00	2.74	26.15	2.62	*<0*.001*
	ZONE 6	29.38	2.61	27.90	3.16	26.28	3.19	*<0*.001*

Correlation analysis between retinal measurements and MS severity (by EDSS) showed a moderate correlation with mean RNFL thickness (r = 0.605; p = 0.043). Particularly in relation to time of CDMS diagnosis, more than 50% of cells showed a mild inverse correlation with the Pearson correlation coefficient. In addition, the RNFL showed a significant moderate inverse correlation with the EDSS baseline (EDSS-0) score and with the EDSS score at 1-year follow-up (EDSS-1) (Fig 4).

### Ganglion cell layer

When compared with controls using the Mann–Whitney *U* test, the RR-MS group presented 35 cells with significant differences (p < 0.001*) (Table 1; Fig 2). The Kruskal–Wallis *H* test revealed several differences versus healthy controls in the CSO RR-MS subgroups (Table 2), and highly significant differences were observed in 5 zones.

*Post hoc* analysis with Bonferroni correction (Table 2; Fig 3, upper) showed significant differences in subgroup CSO-1 versus healthy controls in zones 1 (p < 0.001), 2 (p < 0.001), 5 (p = 0.009), and 6 (p = 0.024). In subgroup CSO-2, significant differences were found in zones 1 (p < 0.001), 2 (p < 0.001), 3 (p = 0.008), 5 (p < 0.001), and 6 (p < 0.001). No differences were found between CSO subgroups at GCL layer level.

In the CDMS subgroups, significant differences (p < 0.001*) were observed in 31 cells (Table 3; Fig 3, lower). When the CDMS subgroups were compared with healthy controls, *post hoc* analysis revealed significant differences with Bonferroni correction in subgroup CDMS-1 in zones 1 (p < 0.001), 2 (p < 0.001), 5 (p = 0.004), and 6 (p = 0.045). Subgroup CDMS-2 showed significant differences in zones 1 (p < 0.001), 2 (p < 0.001), 3 (p < 0.001), 4 (p = 0.009), 5 (p < 0.001), and 6 (p < 0.001). In subgroups CDMS-1 and CDMS-2, significant differences were found between zones 1 (p = 0.036), 2 (p = 0.015), 3 (p = 0.004), and 6 (p = 0.028).

Correlation analysis between mean GCL thickness and MS severity (EDSS) did not identify statistical significance (r = 0.701; p = 0.094). The Spearman test revealed similar results for both the GCL and the RNFL for the CSO and CDMS time point correlations (Table 4); significant mild inverse correlation was found with CDMS, and no correlation was observed with EDSS-0 or EDSS-1 (Fig 4).

**Table 4:** Correlation between retinal nerve fiber layer and ganglion cell layer thickness and time since onset of symptoms, time since clinically definite multiple sclerosis diagnosis, Expanded Disability Status Scale (EDSS) at time of optical coherence tomography (OCT) examination, and EDSS 1 year later. The asterisks mark significant levels, * p < 0.05 and ** p ≤ 0.005. Abbreviations: RNFL, retinal nerve fiber layer; GCL, ganglion cell layer; Pcc, Pearson correlation coefficient; CSO, clinical symptom onset; CDMS, clinically definite multiple sclerosis; EDSS, Expanded Disability Status Scale.

		Years since CSO	Years since CDMS	EDSS (OCT date)	EDSS (+1 year)
		n = 100	n = 99	n = 97	n = 79
**RNFL**	ZONE 1	-0.193	*-0*.288**	-0.232*	-0.317**
	ZONE 2	-0.227*	*-0*.299**	-0.254*	-0.296**
	ZONE 3	*-0*.307**	*-0*.378**	-0.344**	-0.331**
	ZONE 4	*-0*.259**	*-0*.352**	-0.317**	-0.264*
	ZONE 5	-0.173	*-0*.289**	-0.301**	-0.218
	ZONE 6	-0.111	-0.149	-0.101	-0.16
**GCL**	ZONE 1	-0.180	*-0*.268**	-0.109	-0.108
	ZONE 2	*-0*.243*	*-0*.320**	-0.162	-0.201
	ZONE 3	*-0*.283**	*-0*.323**	-0.070	-0.086
	ZONE 4	-0.085	-0.143	-0.001	-0.012
	ZONE 5	-0.198*	*-0*.274*	-0.048	0.012
	ZONE 6	*-0*.258**	*-0*.332**	-0.121	-0.128

## Discussion

The aim of this study was to ascertain whether the OCT Spectralis posterior pole protocol provides precise information about retinal layers usable in early detection and monitoring of multiple sclerosis. This is a novel study; according to our bibliography review, this is the first paper to use the posterior pole protocol with MS patients.

Using the posterior pole protocol, we found that both the GCL and the RNFL showed significant thinning when comparing RR-MS patients with healthy controls. Our results support previous findings in studies that used the classic Spectralis protocols [12, 16–18, 25, 30]. This study, however, provides new results achieved with the posterior pole protocol, which offers the opportunity to analyze larger areas than the classic macular protocols based on ETDRS grid analysis. In addition, the posterior pole protocol improves anatomical determination of the location of the papillomacular bundle. This bundle is very important in MS since previous studies have demonstrated that the papillomacular bundle indicates onset of one of the first anatomopathological processes established in MS, hence its potential value as an early diagnostic biomarker [31].

Considerable reduction in axonal density has been demonstrated in areas of demyelination, but not all axons are damaged to the same extent. Axons with smaller diameters are more vulnerable than those with larger ones [32]. It is known that axons are distributed in the retina in differing proportions depending on their diameter, and that there is a greater density of axons of smaller diameter around the macula and the papillomacular bundle [31, 32].

In our study of patients with early-stage MS, it is precisely this region that exhibits the most notable decline in thickness. These small dimensions have the drawback of being challenging for OCT to measure, potentially making segmentation in areas of thinning less precise.

Traditionally, the peripapillary RNFL has been used to evaluate MS patients [6, 8, 33, 34]. Our results confirm previous findings regarding the significant impact MS has on the GCL, even in early stages of the disease following symptom onset. These results do not diminish the importance of the RNFL as significant correlations were observed in GCL and RNFL thickness. Our results also suggest that the GCL is affected before the RNFL. This was observed in the CSO-1 and CDMS-1 groups, which had zones with significant GCL damage. Meanwhile, zone 5 (inferior-temporal) and zone 6 (superior-temporal) did not exhibit differences in RNFL. In zone 4 (inferior-nasal), while the RNFL was affected the GCL was not affected for the first 5 years in both groups and subgroups.

These results are in accordance with those reported by Green, Ratchford, Pietroboni et al [17, 18, 35]. Additionally, Altan et al. [21] found that, in contrast with the RNFL, GCL thickness seems to have no intra-eye asymmetry in healthy Caucasian subjects. This, in addition to our own findings, gives more consistency to the hypothesis of GCL as a biomarker for MS diagnosis and follow-up.

Regarding the EDSS in our patients, significant correlations were observed in the retinal fiber layer at the time of the OCT scan but not in the GCL; similar findings were made by Shi C et al. [36] and Eslami et al [37]. The reason for this, we believe, is that the EDSS is a parameter without a complete linear progression; it varies with disease episodes (intensity and type). Interestingly, we found areas of correlation between the RNFL and the EDSS 1 year after the OCT examination. Similar results were observed by Montolio et al. [38] and Rothman et al., [39] who found a significant inverse correlation between macular volume and EDSS 10 years later.

Our results suggest that, in MS, the posterior pole protocol detects GCL affectation earlier than changes in the RNFL are detected. Also, the GCL seems to be the layer best correlated with disease duration even when starting from the time of symptom onset. A time lapse between symptom onset and definite MS diagnosis exists and while in some cases this lapse may extend to several years, retinal changes are already established. In addition, our results suggest that the RNFL is a good indicator of disease severity prognosis (EDSS score 1 year later); the greater the macular RNFL thinning, the greater the possibility of disability progression.

The main limitations of this study are as follows: i) It is monocentric and has used a single OCT acquisition system. It would be useful to analyze whether the conclusions of this paper can be generalized to include other conditions, other populations, and even other OCT devices; ii) It is a cross-sectional study. If it included longitudinal patient follow-up, the usefulness of the method in monitoring the disease could be assessed; iii) The time-based allocation of the subgroups could include errors due to the lack of precision in the information on the dates of disease diagnosis and outbreak in the available documents.

In conclusion, wide macular OCT, as well as the posterior pole protocol, are helpful tools with which to diagnose and monitor RR-MS in eyes without a previous history of optic neuritis. Protocols not based on the ETDRS grid allow practitioners to identify individual areas of damage and to detect clusters on retinal layers and so enable accurate follow-up. This protocol could also offer a useful way to predict disability due to the measurement accuracy of the APS system, which defines the papillomacular bundle better than other alternatives and provides greater scan density: 61 scans versus 49 scans under the classic fast macular protocol. Further studies evaluating the capability of this new posterior pole protocol to diagnose early-stage MS in different MS phenotypes and in eyes with a history of optic neuritis would help us to understand the pathophysiology of this disease better.

The greatest advantage of this protocol is that analyzes all the layers of the retina in their actual location rather than drawing conclusions from small analyses obtained using automatic ETDRS analysis. The International Multiple Sclerosis Vision System Consortium (IMVISUAL) [40] acknowledges that data published in recent years demonstrate the effectiveness of OCT in the diagnosis and treatment of MS. The future of this research is oriented toward applying artificial intelligence in the diagnosis and management of neurodegenerative diseases using OCT.

## Supporting information

S1 Dataset (XLSX)
